# Theoretical characterisation of electron tunnelling from granular activated carbon to electron accepting organisms in direct interspecies electron transfer

**DOI:** 10.1038/s41598-022-15606-8

**Published:** 2022-07-20

**Authors:** Rohan Rao, Jing Hu, Po-Heng Lee

**Affiliations:** 1 Department of Civil and Environmental Engineering, Imperial College London, South Kensington Campus, London, UK; 2 Department of Physics, Oxford University, Oxford, UK

**Keywords:** Biophysics, Environmental sciences, Chemistry, Engineering

## Abstract

Direct interspecies electron transfer (DIET) has been identified as an efficient metabolism between symbiotically interacting organisms. One method of DIET uses conductive materials (e.g., granular activated carbon (GAC)) as a medium to shuttle electrons from electron donating organisms (eg., *Geobacter metallireducens*) to electron accepting organisms (e.g., *Geobacter sulfurreducens* and *Methanosarcina barkeri*). Conductive materials such as GAC, become negatively charged in DIET processes due to reduction by electron donating organisms. This high excess electron density in GAC leads to quantum tunnelling of electrons being a significant electron transfer mechanism for DIET. Thus, a theoretical model obeying the Wentzel–Kramers–Brillouin (WKB) approximation and Fermi–Dirac statistics was developed and simulated. In the model, the electron tunnelling transfer barrier was described by an effective rectangular barrier. The result of our 1D tunnelling simulations indicates that within 29.4 nm of the GAC, tunnelling can sufficiently supply electrons from GAC to *G. sulfurreducens* and *M. barkeri*. The phenomenon of tunnelling may also have significance as a stimulant of chemotaxis for *G. sulfurreducens* and other electron accepting microbes when attempting to adsorb onto GAC. This study sheds light on quantum tunnelling’s significant potential in both bacterium and archaeon DIET-centric processes.

## Introduction

Direct interspecies electron transfer (DIET) has raised great interest due to its more efficient mechanism in methanogenesis and sulfidogenesis kinetics^1–3^. Currently, DIET has been reported between methanogens and exoelectrogens. The electron exchange under such association is proposed through (1) the cytochrome c or pili located in their outer cell surface of exoelectrogens^1,4^ and (2) conductive materials (e.g., biochar, carbon cloth, graphene, ferrihydrite, stainless steel and granular activated carbon (GAC))^5^. The latter (conductive materials) have a longer-range electron transfer mechanism over the former (electrically conductive pili) among the DIET partners^4^ since the conductive materials can serve as electron transfer media for symbiotically interacting DIET partners across hundreds of nanometres. In this paper, we focus on two situations where GAC acts as an electron transfer medium in DIET: The first is where *Geobacter metallireducens* and *Geobacter sulfurreducens* play the role of electron donating- and accepting-microorganism, respectively^6^. The second is where *G. sulfurreducens* is the donor once more but *Methanosarcina barkeri* acts as the electron acceptor-microorganism^3^. The mechanism for the donation of electrons from *G. metallireducens* to GAC has been well studied. A study from Rotaru et al.^7^ indicates that outer membrane c-type cytochromes and pili are responsible for the donation of electrons to GAC from *G. metallireducens*. However, the mechanism for electron acceptance from conductive materials to *G. sulfurreducens* and *M. barkeri* has had little exploration.

For *G. sulfurreducens*, it had been previously hypothesised that pili acted as the pathway for electrons to be accepted from conductive materials but this was contradicted by Ueki et al.^8^, who found that the inhibition of the piliA genus had no effect on the ability of *G. sulfurreducens* to accept electrons from a cathode. Kracke et al.^9^ found when the periplasmic c-type cytochrome gene GSU3274, which codes for the PccH protein^10^ was inhibited, *G. sulfurreducens* could not receive electrons from a cathode. This evidenced that PccH is critical to *G.Sulferreducens’* electron accepting pathway within the periplasm but the study does not comment on the primary electron acceptors for *G. sulfurreducens*. Recently, Heidary et al.^11^ have found evidence that iron oxide (FeO_x_) nanoparticles act as the primary electron accepting sites for *G. sulfurreducens*. These iron oxide particles come about from the breakdown of Fe-cytochromes and cover the surface of *G. sulfurreducens*. This suggests that FeO_x_ nanoparticles are the ‘outer surface electron accepting sites’ (OSEAS) for *G. sulfurreducens*. As for *M. barkeri* in its normal conditions, it is surrounded by a mesh of an expolysacchiride known as methanochondroitin (MC) above the S-layer^12^. Rowe et al.^13^ found that electron uptake took place from cathodes due to electron transfer through ‘Direct Contact’. It has been shown that MC is a conductor of electrons^14,15^ and as such can be modelled as the OSEAS for *M. barkeri*. Therefore the ‘Direct Contact’ process strongly suggests that *M. barkeri* have MC as their OSEAS, acting as electron acceptors from conductive materials during DIET*.*

The natural question of how these electrons get transported from GAC to *G. sulfurreducens*’ outer surface iron oxide particles (or to *M. barkeri*’s MC layer) arises. The large negative charge density on the GAC, due to *G. metallireducens* reduction, in combination with the small distance scales between the electron acceptor and GAC provide an ideal environment for quantum tunnelling of electrons to the OSEAS to occur. Quantum tunnelling enables electron transfer through a potential barrier although it possesses insufficient (activation) energy. Quantum tunnelling through methanophenazine (MP), a cellular conductive substance, in the electron transport chain of *Methanosarcina acetivorans* C2A has been evidenced. Duszenko and Buan found that, at high electron carrier density, high MP content induces tunnelling kinetic rates^1^. This likely occurs in GAC-induced DIET methanogenesis through the tunnelling of electrons in aqueous solution near the electrodes (GAC) to which voltage is applied from electron exporting bacteria, such as *Geobacter* spp. Indeed, Shin et al.^16,17^ reported low threshold values of substrate utilization by GAC-induced DIET-methanogenesis in a lab- and pilot-scale investigation, resulting in “non-classical” faster kinetics rates. This implies that quantum tunnelling could be possible in the context of electron accepting organisms within DIET, so it is important to investigate whether it contributes a significant electron flux from GAC to electron accepting organisms. This paper aims to theoretically model the magnitude and range of this long-range tunnelling flux obeying the Wentzel–Kramers–Brillouin (WKB) approximation and Fermi–Dirac statistics. The WKB approximation enables the estimation of bound state energies in quantum systems and can be used to find tunnelling rates through potential barriers. Fermi–Dirac statistics characterises the probability of an electron occupying a specific energy level within a free electron model of a conductive material. The model thus calculates an effective rectangular tunnelling barrier by averaging over the spatially varying and temporally varying potential barrier between GAC and the electron accepting organism. The results of the model shed light on the potential of electron tunnelling to be a significant mechanism by which DIET from conductive materials to electron accepting organisms is achieved.

## Methodology: model development

In this analysis, we model the process of electron tunnelling from GAC through an aqueous solution to OSEAS, using the analysis from Simmons^18^ and Guo et al.^19^ (A1). Simmons gives the equation for the current density, $J$,of electrons that tunnel out of a metal that obeys Fermi–Dirac statistics:

1 $$J = J_{0} \left( {\overline{\phi }{\text{exp}}\left( { - A\overline{\phi }^{\frac{1}{2}} } \right) - \left( {\overline{\phi } + eV_{eff} } \right){\text{exp}}\left( { - A\left( {\overline{\phi } + eV_{eff} } \right)^{\frac{1}{2}} } \right)} \right)$$

2 $$A = \frac{{4\pi \beta {\Delta }s}}{h}\left( {2m_{e} } \right)^{2}\ \ \ \ \ \ J_{0} = \frac{e}{{2\pi h\left( {{{\beta \Delta }}s} \right)^{2} }}\ \ \ \ \ \ \overline{\phi } = \frac{1}{{{\Delta }s}}\mathop \smallint \limits_{0}^{s} \phi \left( x \right)dx$$

where $\beta$ is a correction factor that approximates to one in the limits of the accuracy we require $\left( {\beta \approx 1} \right)$. ${\Delta }s$ is the distance between the fermi level of the electron in the metal and the electron level in the OSEAS. ‘$s$’ is the separation between the GAC surface and the electron accepting organism surface. $\overline{\phi }$ is the mean potential barrier between the GAC and the OSEAS and $V_{eff}$ is the effective voltage between the GAC and the OSEAS. $e,\pi$,$h$ are the usual values of the electronic charge, Pi and Planck’s constant respectively. To find the electron flux, *J*, from GAC to the electron accepting organisms as a function of separation, $s$, the values of $V_{eff}$ and $\overline{\phi }$ need to be ascertained.

Simmons’ calculation is for electron tunnelling fluxes between two metals through an insulator, but his calculations can be generally extended to our case of tunnelling from GAC to OSEAS on the surface of *G. sulfurreducens* and *M. barkeri* depending on two conditions being satisfied: 1) the limits of the Wentzel–Kramers–Brillouin (WKB) approximation being valid and 2) GAC obeys Fermi–Dirac statistics. For the first condition to hold, our model must have a slowly varying potential barrier that spatially varies on the scale of the de Broglie wavelength of the electron or larger. The de Broglie wavelength of the highest energy electrons in GAC (in other words the Fermi wavelength) can be found using the Fermi velocity:

3 $$\lambda_{de broglie} \sim \frac{h}{{\left( \frac{3}{5} \right)^{0.5} m_{e} v_{f} }}\sim 9.38 \times 10^{ - 10} \;{\text{m}}$$

where we have taken the Fermi velocity to be ~ 10^6^ ms^−1^ for GAC^20^. The potential barrier varies on the same scale as the average distance between adjacent solvation electron states, which are electron states with binding energy minima within water^21,22^. Solvation states have been analysed extensively through the cavity model, which gives an approximate length scale separation between solvation states of ~ 4.6 × 10^−10^ m^22^. The De Broglie wavelength and distance between solvation states are of the same order of magnitude and so the first condition for the WKB approximation is satisfied.

For the second condition to hold, it is necessary for GAC to be treated as an object that obeys Fermi–Dirac statistics before Simmons’ analysis can be used. GAC has a turbostratic carbon structure with hexagonal and pentagonal quasi-crystalline regions of carbon atoms within its macrostructure^23,24^. Within this macrostructure, GAC can be modelled as being composed of domains of conductive graphite that have a maximum lateral dimension of 2–5 nm^23^. Therefore, for the purposes of electron tunnelling we can model the energy distribution of electrons within GAC as each having the same electronic band structure as graphite. This assumes that electrons in each graphitic domain do not see the potential from other domains due to screening. Graphite’s electron tunnelling rates can be treated with the same equation as that of a Fermi–Dirac obeying metal if an effective mass for the electron in graphite is used^25^ to account for Graphite’s electronic band structure. Therefore, we can use the analysis of Simmons with a correction factor for the effective electron mass^25^.

4 $$m_{eff} = \frac{{2\gamma_{1} }}{{v_{f}^{2} }}$$

where $m_{eff}$ is the effective mass of the electron in graphite^25^, $\gamma_{1}$ is the empirically found nearest neighbour interaction between atoms of the same type, $v_{F}$ is the Fermi velocity of electrons in graphite at the Dirac point and $m_{e}$ is the rest mass of a free electron. Brandt et al.^26^ have found $\gamma_{1} = 39e$ where *e* is the magnitude of the electronic charge of an electron. As before $v_{F} \approx 10^{6}$ for graphite. Therefore

5 $$m_{eff} = \frac{{2\gamma_{1} }}{{v_{f}^{2} }} \approx \frac{{2 \times 0.39\;{e}}}{{\left( {10^{12} } \right)}} = 0.14\;m_{e}$$

**Figure 1:**
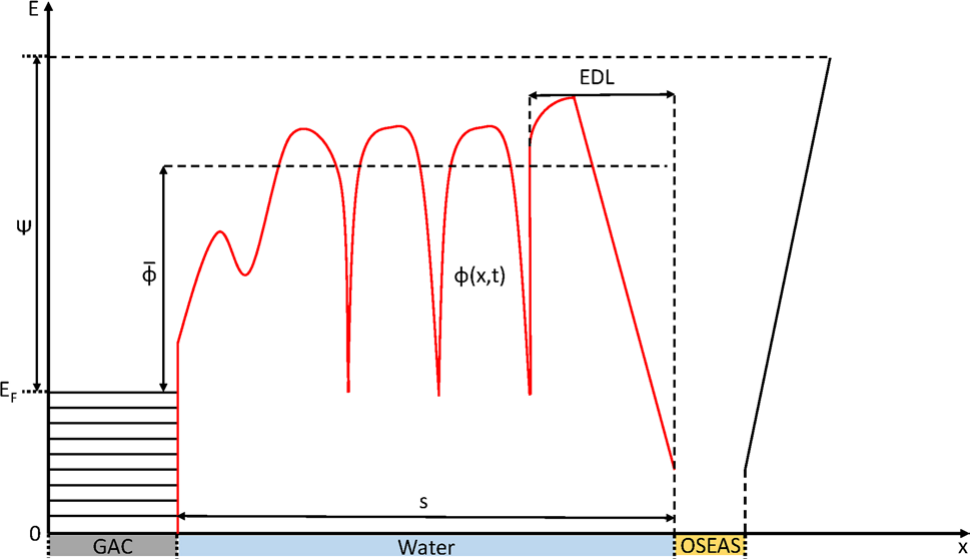
General 1-dimensional potential diagram for an electron tunnelling from GAC to the OSEAS of the electron receiving organism. Energy (E) on the y axis and distance (x) on the X axis. ${\Psi }$ is the work function of graphite in a vacuum. The electric double layer (EDL) contribution from the negative charge of the electron accepting organisms outer surface causes the mean potential barrier $\left( {\overline{\phi }} \right)$ faced by the electron to increase. Troughs in the potential barrier, $\phi \left( {x,t} \right),$ which extends through the water domain, are caused by electron solvation states in the water. The GAC domain shows characteristic energy bands that the electrons occupy. The OSEAS domain shows a potential well that is energetically favoured for the electron. ‘*s*’ is the separation between GAC and the OSEAS.

This effective mass for the electron can now replace the rest mass of the electron in Eq. (1) (assuming GAC has an isotropic band structure). The electron tunnelling flux through water is displayed as a 1-dimensional potential energy diagram in Fig. 1.

We have assumed in this model that the potential barrier from water is static. The potential varies over the time scale the H_2_O molecules diffuse a distance equal to their own length which is ~ 10 ps (A2) so our model averages over a time that is much larger than 10 ps to ensure the assumption of a static potential holds. These solvation states are represented by the potential valleys in the water domain of Fig. 1 and they have a vertical binding energy of between 3.03–3.27 eV^19^. Gold has its work function lowered from its dry value of Ψ_gold_ = 5.1–5.47 eV^19^ to Ψ_gold_^ʹ^ = 2.26 ± 0.1 eV^19,27^ (the prime indicates a submerged value rather than a dry value) when submerged under water. Guo and Mackenzie^19^ showed that electrons tunnelling from a gold electrode to hydroxide ions faced a potential barrier at the gold electrode of φ_gold_ = 0.26 ± 0.01 eV due to the intermediate solvation states in bulk water.

Similarly Leenaerts et al.^28^ showed computationally that, like gold, many layers of graphene (in other words graphite) have their work function lowered down to Ψ'_graphite_= 2.1 eV, when the graphene is charged and a dipole layer of water molecules is placed on their surface. This is much lower than the work function of dry graphite: Ψ_graphite_ = 4.5eV ^25^. As the GAC in our system is highly negatively charged by *G. metallireducens*, submerged GAC can also be modelled to have a submerged work function of Ψ'_graphite_= 2.1 eV. The corresponding lowering of the average potential barrier, $\overline{\phi }$, of tunnelling from GAC is therefore extremely significant as a result of these solvation intermediate states. Given that the work function of submerged graphite and gold is so similar we can now make the approximation that the potential barrier height an electron faces from graphite or gold is scaled by the ratio of work functions under water: Ψ'_graphite_ and Ψ'_gold_. This approximation is valid in the regime that the potential barrier heights of graphite or gold under water are proportional to the work functions of the materials under water:

6 $$\varphi { }_{1} \approx \frac{{{\Psi }_{graphite}^{{\prime }} }}{{{\Psi }_{gold}^{{\prime }} }}\varphi { }_{gold} = 0.24 \pm 0.1\;{\text{eV}}$$

This value for $\varphi { }_{1 }$ has not yet considered the electric double layer surrounding the electron receiving organism due to the negative surface charge of the *M. barkeri* surface layer and the *G. sulfurreducens* lipid bilayer. We will now account for this through the following the equations from Simmons.

7 $$\overline{\phi } = \overline{\phi }_{1} + \overline{\phi }_{2} \left( s \right)$$

8 $${\Delta }s \approx \frac{{s\overline{\phi }_{1} }}{{eV_{eff} }}\;\overline{\phi }_{1} = \left( {\varphi { }_{1} } \right)/2 = 0.12 \pm 0.05\;{\text{eV}}$$

9 $$\overline{\phi }_{2} \left( s \right) = \frac{1}{{{\Delta }s}}\mathop \smallint \limits_{0}^{s} V_{organism\;EDL} \left( x \right)dx = \frac{G\left( s \right)}{{{\Delta }s}}$$

**Figure 2:**
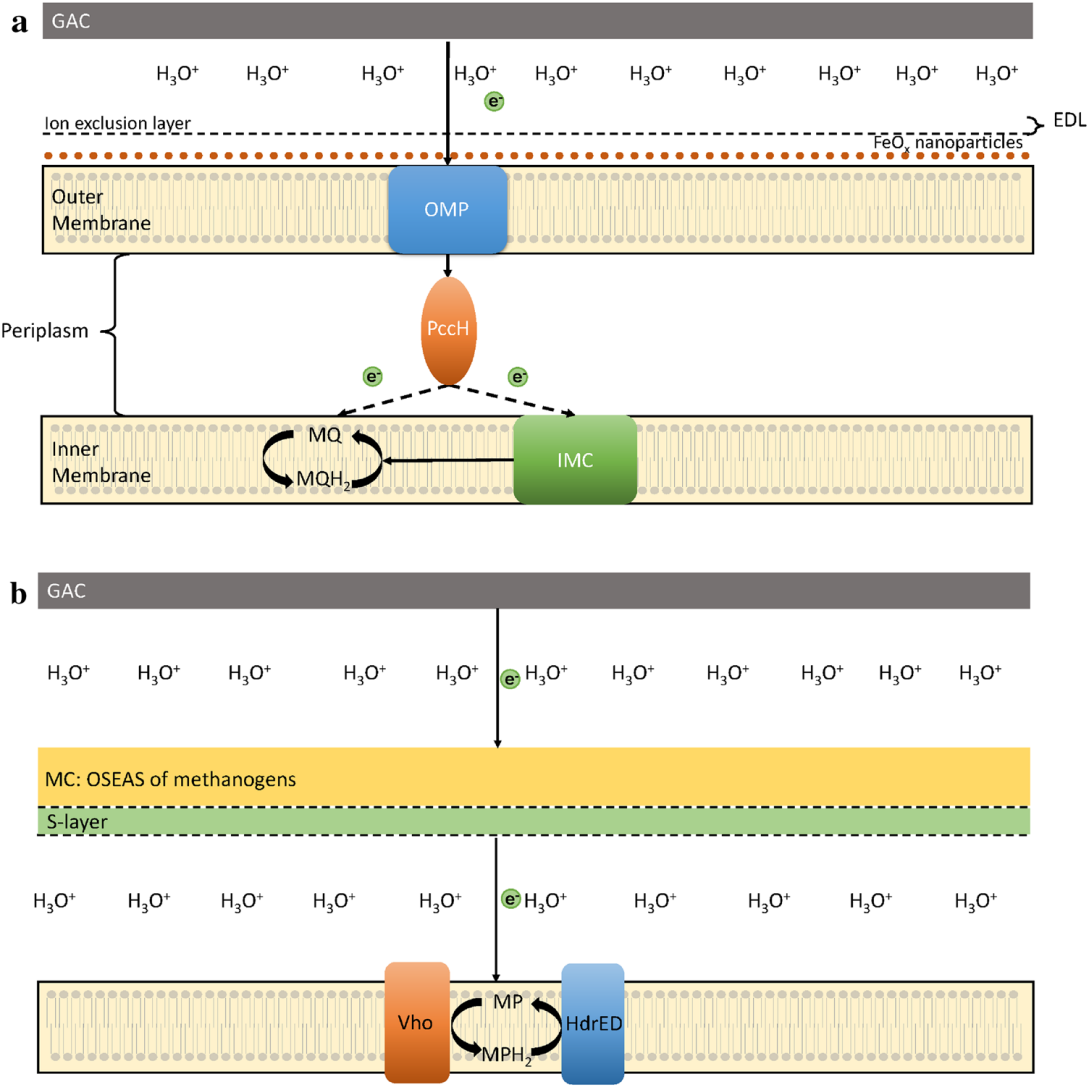
(**a**) Diagram of the electron tunnelling pathway from GAC to *G. sulfurreducens*. The lipid bilayer outer membrane is covered by FeO_x_ nanoparticles that act as the hypothesised OSEAS. These electrons are then shuttled from the FeO_x_ nanoparticles through a hypothesised outer membrane protein (OMP) to the periplasmic cytochrome c (GSU3274; PccH) further to menaquinone (MQ) and/or inner membrane cytochrome (IMC). Note: Hydrogenated MQ (MQH_2_). (**b**) Diagram of hypothesised electron tunnelling pathway from GAC to *M. barkeri*. In our model, electrons tunnel from GAC to the conductive MC layer surrounding *M. barkeri* before being transported through the S layer of the archaea to methanophenazine (MP) in the lipid bilayer. A membrane-bound (F420 non-reducing) hydrogenase (Vho) and a membrane-bound heterodisulfide reductase (HdrED).

$\overline{\phi }_{1}$ represents the average potential barrier contribution from the solvation states within the water and the electric double layer (EDL) from the GAC. The EDL refers to the stern layer and diffuse layers of ions and H_2_O molecules surrounding a charged object in an aqueous solution. The stern layer consists of adsorbed water molecules and ions, whereas the diffuse layer is composed of water molecules and ions whose arrangement is governed by the Coulomb force and stochastic thermal forces ^19,29^. The addition of the factor, $\overline{\phi }_{2}$ , accounts for the contribution to the average potential barrier from the EDL of the electron receiving organisms as the electron receiving organisms have charged outer surfaces. Figure 2a shows a scheme of the electron tunnelling pathway from GAC to *G. sulfurreducens*:

The EDL caused by the outer membrane of *G. sulfurreducens* increases the potential barrier that the electron faces due to the negatively charged head groups of the lipid bilayer membrane (Fig. 2a). Peitzsch et al.^30^ found the potential of an electric double layer due to a lipid bilayer using the Poisson-Boltzmann equation and found their results to be in close agreement with the Guoy-Chapman model for the EDL. Above the headgroups of the lipid bilayer exists an ion-exclusion layer that is $2\dot{A}$ thick, so the EDL potential has its origin (x = 0) at the surface of the ion-exclusion layer, not the surface of the lipid bilayer headgroups. We can use a high potential limit of the Poisson-Boltzmann equation to find how the potential barrier of the EDL varies with distance. The FeO_x_ nanoparticles will be assumed to extend through the ion exclusion layer ($2\dot{A}$ thick) and be uncharged. The Poisson-Boltzmann equation for a large potential (e $\left| V \right| > k_{b} T_{room} \approx 0.03eV)$ as a function of distance from the ion exclusion layer takes the form shown by Langner et al.:^31^

10 $$V_{organism\;EDL} \left( x \right) = \frac{{2k_{B} T}}{e}\left( {\ln \left( {\frac{{1 + \alpha \exp \left( { - \kappa x} \right)}}{{1 - \alpha \exp \left( { - \kappa x} \right)}}} \right)} \right)$$

where

11 $$\alpha = \frac{{\exp \left( {\frac{{eV_{organism\;EDL} \left( {x = 0} \right)}}{{2k_{b} T}}} \right) - 1}}{{\exp \left( {\frac{{eV_{organism\;EDL} \left( {x = 0} \right)}}{{2k_{b} T}}} \right) + 1}}$$

and $$\frac{1}{\kappa } = {\text{Debye length }}$$.

**Figure 3:**
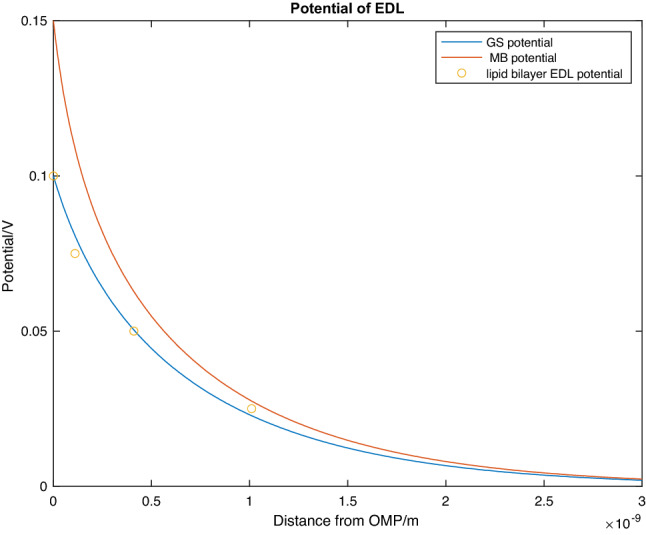
Plot of potential through the EDL against distance from the outer membrane proteins (OMP). The *G. sulfurreducens’* (GS) EDL potential curve (blue) was calculated from Eq. (9) and was fit against the data (orange dots) from Peitzsch et al.^30^. The *M. barkeri* (MB) curve was then found using the same method using the results from Li et al.^33^.

**Figure 4:**
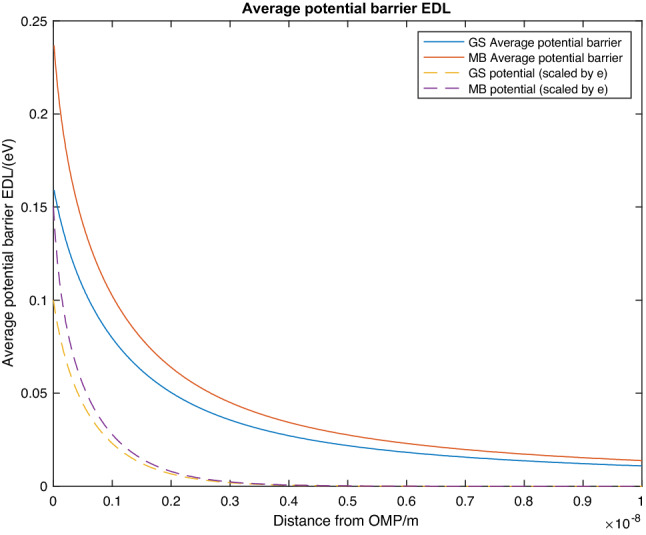
Plot of the average potential barrier from the EDL: $\overline{\phi }_{2} \left( s \right) = G\left( s \right)/s = \mathop \smallint \limits_{0}^{s} V_{organism\;EDL} \left( x \right)dx/s$ against distance(*s*) from the accepting organism for *G. sulfurreducens* (GS: solid blue) and *M. barkeri* (MB: solid orange). The dotted lines show the original potentials,$V_{organism EDL} \left( s \right)$, in Fig. 3

The potential barriers from the EDL of *G. sulfurreducens* and *M. barkeri* are plotted in Fig. 3. The Debye length is evaluated by fitting the curve to the data provided by Peitzsch et al.^30^ in Fig. 4. Peitzsch et al.^30^ gives the result:

12 $$V_{GS\;EDL} \left( {x = 0} \right) = 0.1\,{\text{V}},\;\frac{1}{\kappa } = 0.83\;{\text{nm}}$$

Equation (12) and (10) thus gives the behaviour of the potential from the EDL from *G. sulfurreducens*.

The OSEAS of *M. barkeri* is the entire MC layer surrounding the organism, which we will assume to be neutrally charged and doesn’t affect the potential of the EDL (Fig. 2b). (Sowers et al.^11^ have found that the positively charged N-acetyl-D-galactosamine in the MC layer causes the charge of the surface to be net positive but we will neglect the effect of this charge as the MC mesh contains many hydration shells that screens its contribution to the EDL). Therefore, we will account for the EDL from *M. barkeri* by considering the negatively charged S-layer of *M. barkeri* only and neglect the effect of MC on the EDL. Figure 2b shows a schematic of the electron tunnelling pathway from GAC to *M. barkeri.* The S-layer contains a negative surface charge^32^ and causes an EDL potential with the following boundary conditions^32^.

13 $$V_{MB\;EDL} \left( {x = 0} \right) = 0.15\,{\text{V}}.\;\frac{1}{\kappa } = 0.82\;{\text{nm}}$$

Now that the boundary conditions and equations for the potential from the EDL of both *M. barkeri* and *G.Sulfurreducens* is known (Eqs. (10), (12) and (13)), Fig. 3 shows the EDL potential for both organisms. The behaviour of the average potential barrier of the EDL as a function of the distance from the organism is approximately an exponential decay illustrated in Fig. 4. The contribution of the EDL has been accounted for (i.e. $\overline{\phi }_{2}$ is known) , the remaining unknown parameters in Eq. (1)$\left( {V_{eff} \;a{\text{nd}}\;{\Delta }s} \right)$ are required to calculate the electron tunnelling rate. The potentials from the EDL of *M. barkeri* and *G. sulfurreducens* can now be used to calculate the contribution of the EDL to the average potential barrier. Using Eq. (9), the contribution from the EDL of the two organisms to the average potential barrier was calculated numerically (Fig. 4).

*G. Metallireducens* can reduce GAC to its maximum capacitance of 40 nF^34^ via its extracellular electron transfer pathway that falls from a potential of − 0.38 V^35^. We can see from the work of Caizan et al.^36^ that the steady state potential on the GAC particles is equal to that of the reduction potential of the metabolic reaction carried out by the electron donating organism. Therefore, we can say that the steady state potential on the GAC particle is also − 0.38 V. As such, we can model a typical GAC particle in our system as a cathode at a potential − 0.38 V. The negative charge in the GAC forms an EDL on the GAC surface in the aqueous solution^16^. We have accounted for the EDL contribution from the GAC by absorbing it into our mean potential barrier $\overline{\phi }_{1}$ so it has been accounted for. As such, the parameters for $V_{eff}$ are

14 $$V_{eff} = V_{GAC\;surface} = 0.38\,{\text{V}}$$

Simmons^17^ gives ${\Delta }s$ in the high voltage limit as

15 $${\Delta }s \approx \frac{{\varphi { }_{1} s}}{{eV_{eff} }} = \frac{0.24}{{0.38}}s = 0.63\,{\text{s}}$$

with $\overline{\phi }$ and $V_{eff}$ known, the flux, J, is known as a function of separation,$s$. The rate of electrons entering the receiving sites varies with s in the following equation:

16 $$R\left( s \right) = \frac{{J\left( s \right)A_{eff organism} }}{e}$$

$R$ is the rate (s^−1^) of electrons transferred from the GAC to a single organism and $A_{eff}$ is the surface area of OSEASs on the surface of the electron receiving organism. The area of the electron accepting proteins is calculated using the following formulae:

17 $$A_{eff\,organism} = A_{projection} \times A_{{{\text{OSEAS}}}} \times \rho_{{{\text{OSEAS}}\,population}}$$

where $A_{eff\,organism}$ is the effective area of electron accepting sites on the microorganism. $A_{{{\text{OSEAS}}}}$ is the electron accepting area of the OSEAS, $A_{projection}$ is the area of the organism facing the GAC and $\rho_{{{\text{OSEAS}} population}} \left( {m^{ - 2} } \right)$ is the population density of the OSEAS.

As described above, recent studies on cathodic growth of *G. sulfurreducens* have shown evidence that iron oxide particles act as redox acceptors for electrons on the cell wall of *G. sulfurreducens*^11^*.* Therefore, we hypothesise that these FeO_x_ nanoparticles are the electron accepting sites on the surface of *G. sulfurreducens* due to the recent studies and the fact that the work of Kracke et al.^9^ has ruled out OmcZ, OmcS and OmcT as potential electron accepting c type cytochromes for *G. sulfurreducens*. We found the dimensions of these FeO_x_ nano particles to be 2–4 nm in length and 0.097 nm in width^11^. In their work they show that a crystalline structure of these FeO_x_ nanoparticles covers the entire surface of the *G. sulfurreducens* with an approximate spacing of 0.058 nm between nanoparticles. Therefore, the product: $A_{FeOx} \times \rho_{FeOx\;population} \sim \frac{0.097}{{0.097 + 0.058}} = 0.63$ shows that approximately 63% of the surface is covered in these iron oxide crystals. The shape of *G. sulfurreducens* is approximately a cylinder with radius of 0.3 μm and length of 1 μm^28^, so $A_{projection\;GS} { } = \left( {0.3\;{\mu m} \times 2} \right) \times 1\;{\mu m} = 0.6\;{\mu m}^{2}$^37^. This gives $A_{eff\;GS} = A_{projection\;GS} \times A_{FeOx} \times \rho_{FeOx\;population} \approx 0.4\;{\mu m}^{2}$.

In DIET using GAC in natural conditions, *M. barkeri* is surrounded by an exopolysaccharide layer of MC. This is a conductive complex that acts as the OSEAS for *M. barkeri*. MC surrounds the entire *M. barkeri* but only extends out by ~ 2nm^12^ beyond the outer membrane of and so *M. barkeri* has an effective area of electron acceptance that can be approximated as equal to the surface area of *M. barkeri* itself. The dimensions of a typical spherical *M. barkeri* in a liquid medium are approximately 0.5 μm in radius^38^. Therefore, $A_{projection\;MB} = \pi \left( {0.5\;{{\mu m}}} \right)^{2} \approx 0.8\;{\mu m}^{2}$ and $A_{eff\;MB} = A_{projection\;MB} \approx 0.8\;{\mu m}^{2}$.

## Results and discussion

**Figure 5:**
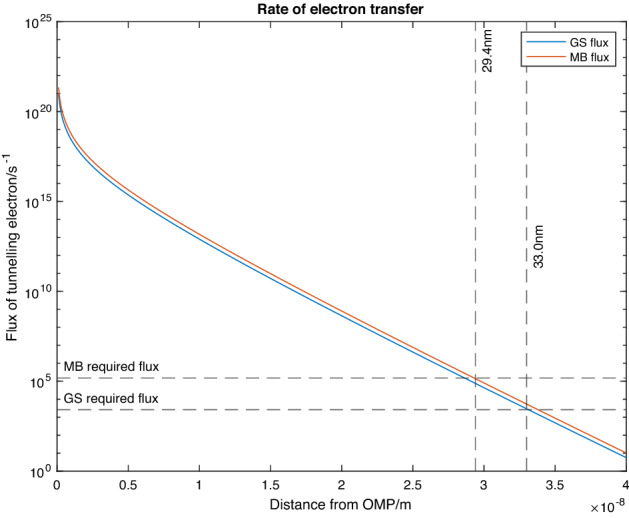
Displays the flux of tunnelling electrons, *R(s),* as a function of separation, *s,* between the electron accepting organism and GAC. *G. sulfurreducens* (blue line) has a slightly higher rate of tunnelling than *M. barkeri* due to its higher area of acceptance and lower EDL potential barrier. Both organisms can accept sufficiently large populations of electrons by tunnelling within 33.0 nm of GAC.

Figure 5 plots the results of Eq. 16 for both *M. barkeri* and *G. sulfurreducens*. The figure shows that there is a faster than exponential drop in electron flux for *G. sulfurreducens* and *M. barkeri* for seperations of 0–5 nm. For seperations larger than 5 nm the electron flux drops exponentially for both organisms. This exponential dependence means that the value of $A_{eff }$ is quite insignificant in determining the range of quantum tunnelling as unless its value is different by many orders of magnitude (which is unlikely within unicellular organisms), the range is almost entirely determined by the value of the potential barrier height.

It is now necessary to ascertain what electron flux *M. barkeri* and *G. sulfurreducens* require for their basic metabolic rate. Using the metabolic chemical equation (the Sabatier reaction) for *M. barkeri*:

18 $${\text{CO}}_{{2}} + {\text{4H}}_{{2}} + {8}e^{ - } \to {\text{CH}}_{{4}} + {\text{2H}}_{{2}} {\text{O}}$$

The energy released in each reaction is 164 kJ/mol of CH_4_^39^. This means 2.72 × 10^–19^ J of energy is released in every reaction and 8 additional electrons are required for every reaction. Archaea, such as *M. barkeri*, have a mean metabolism of 10 W/kg^40^. The dimensions of a spherical *M. barkeri* in a liquid medium are approximately 0.5 μm in radius^38^ and have a density of approximately 1000 kg/m^3^. Therefore, each archaeon has a mass of ~ 5.2 × 10^–16^ kg and a power output of ~ 5.2 × 10^–15^ W. Therefore, there are approximately $\frac{{5.2 \times 10^{ - 15} { }}}{{2.72{ \times }10^{ - 19} }} = 19000$ reactions per second, necessitating an influx of 1.5 × 10^5^ electrons per second. This value of electron flux corresponds with 29.4 nm on Fig. 5, suggesting that if *M. barkeri* is within a separation 29.4 nm of GAC, it could potentially accept sufficient electrons from quantum tunnelling to sustain its basic metabolic rate.

Using a similar calculation but this time for *G. sulfurreducens*: The metabolic equation for *G. sulfurreducens* is:

19 $$fumerate + FADH_{2} \to succinate + FAD,E^{0} = 0.21\,{\text{V}}$$

and the reversible half reaction equations have the following form

20 $$fumerate + 2H^{ + } + 2e^{ - } \to succinate,E^{0} = 0.03\,{\text{V}}$$

21 $$FAD + 2H^{ + } + 2e^{ - } \to FADH_{2} ,E^{0} = - 0.018\,{\text{V}}$$

The change in Gibbs free energy per mole of product for Eq. (19) is Δ*G* =  − *nFE*° =  − 2 × 96,500 × 0.21 = 40.52 kJ/mol of succinate/fumarate (n is the number of electrons in the reaction and F is faradays constant). Therefore 6.73 × 10^−20^ J are released per reaction. We find that given the mean metabolism for proteobacteria (such as *G. sulfurreducens*) is 0.3 W/kg^40^. *G. sulfurreducens* has cylindrical radius, length, mass, and power of 0.3 μm, 1 μm^37^, 2.8 × 10^–16^ kg, and 8.5 × 10^–17^ W, respectively (again a density of 1000 kg/m^3^ is assumed ). Therefore, there are approximately $\frac{{{ }8.5 \times 10^{ - 17} }}{{6.73{ \times }10^{ - 20} }} = 1300$ reactions per second, resulting in a required influx of 2600 electrons/second. This corresponds with a value of 33.0 nm which is only 11% off the 29.4 nm result for *M. barkeri*. This indicates that quantum tunnelling could be a significant long-range electron transfer mechanism when *G. sulfurreducens* is within the range of 33.0 nm of the GAC.

The result of our tunnelling simulations indicates that within 33.0 nm of the GAC, *G. sulfurreducens* can accept a significant amount of electrons from tunnelling. We hypothesise that it is highly likely that the high electron acceptance rate within the 33.0 nm region will act as a stimulant to *G. sulfurreducens* to move towards the GAC through chemotaxis. This may explain one of the contributory mechanisms for how *G. sulfurreducens* congregates rapidly on GAC particles when undergoing DIET with *G. Metallireducens*. The electron accepting *Shewanella putrefaciens* has had its electron accepting chemotaxis mechanism heavily studied and its mechanism for chemotaxis towards high electron densities was observed^41^. We hypothesise that *G. sulfurreducens* has a similar chemotaxis mechanism when in the vicinity of GAC during DIET with *G. Metallireducens*. *S. putrefaciens* may similarly use quantum tunnelling as a method of detecting nearby charged objects to accept electrons so this hypothesis can be applied to all electron accepting organisms capable of Chemotaxis. 33 nm is much larger than the typical ranges of electron tunnelling ~ 0.1–1 nm (as seen in scanning tunnelling microscopy) which is because the decrease in the average potential barrier provided by the electron solvation states leads to an exponential increase in tunnelling range. *M. barkeri*, on the other hand, has not yet been found to be motile, so the long range electron transfer to *M. barkeri* cannot lead to chemotaxis as the adhesion of *M. barkeri* to GAC is purely due to Van Der Waals (VDW) forces and DLVO forces^42^. However, quantum tunnelling still remains relevant as a method of long-range electron transport used by *M. barkeri* that contribute to its ability to accept electrons from cathodes within electron acceptance ranges of 29.4 nm. This model has many assumptions and associated errors within it but has value in affirming at which scales of distance Quantum mechanisms must be considered in aqueous biological processes such as DIET. These simulations have been limited by lack of knowledge of the surface charge of the S-layer and MC layer on *M. barkeri* and this can be rectified by having further study on this area.

It has been found that MC may also contain much better conductive electron pathways within the exopolysaccharide network^14^ and so it may be that these embedded pathways within the MC mesh, contribute a significant proportion of the OSEAS for *M. barkeri*. This does not change the conclusions of this paper as even if the scale factor for the area of electron acceptance assumed in this paper was decreased by a factor of 10 this would only make the critical distance change by 2–3 nm, so using these embedded pathways, or the MC mesh itself, as the electron acceptors gives the correct length scales of tunnelling. As GAC’s work function is dependent on its surface topology^43^, hotspots of tunnelling in regions that have the lowest work function will exist, as these locations will have lower average potential barriers between them and the electron receiving organisms. Further characterisation of conductive materials to induce quantum tunnelling in microbes and other biological systems would advance our understanding and its further applications in surpassing the classical biochemical limitation.

This model has assumed that only electron receiving sites on the geometric shadow of a bacterium can receive electrons from the GAC. The tunnelling rates to sites on the side of the organism furthest from the GAC surface is negligible due to the huge potential barriers within the cell. Further investigation is necessary to explore the exact structure and population density of the electron accepting OSEASs. For example, appropriate gene edited mutants in order to determine the relevant protein complexes required for electron acceptance and then a possible tagging procedure using immunogold labelling or fluorescent labelling techniques would be beneficial. Furthermore, we have assumed that the mean potential barrier of GAC under water can be approximated using that of gold due to their similar work functions under water, but this needs further verification. Additionally, in our model we have assumed that we can use a mean potential barrier to quantise the effect of the EDL by taking the average of it but this may underestimate the actual impact of the EDL as the shape of the EDL has a large gradient. Further experiments could include analysing the electron tunnelling rates from GAC to the electron accepting microorganisms using electron tunnelling microscopy in solution to validate experimental values with this model.

Different materials can be fit into the developed model in this study to aid this quantum tunnelling effect by using materials with the minimum work function available for any biochemical reactions of interest. For example, Duszenko and Buan^1^ reported the low MP content in *M. barkeri* Fusaro has tunnelling potential in the range of 1–1.5 nm. However, with GAC or other conductive material applied, electron tunnelling in *M. acetivorans* can be expanded up to 33 nm. With the material-induced electron tunnelling, this may increase the rate of DIET for efficient energy extraction from wastewater. Similarly, different aqueous solutions with different ion concentrations could be tested to observe how the EDL of the organism and the GAC could be altered to further increase the tunnelling range for DIET.

## Conclusion

The result of 1D tunnelling simulations indicates that both *Geobacter sulfurreducens* and *Methanosarcina barkeri* could potentially accept sufficient electrons via quantum tunnelling to sustain their basic metabolic rate within 29.4 nm of GAC. Furthermore, this tunnelling phenomenon may be applied to all electron accepting organisms (e.g., *G. sulfurreducens* and *M. barkeri*) capable of chemotaxis when gathering on GAC. This study shows the potential mechanisms of electron tunnelling in DIET, facilitating its applications in energy recovery from wastewaters.

## Supplementary Information

Supplementary Information.

## Data Availability

This study is a theorical simulation. No data are used in the simulation. The assumption and code used are described in the manuscript and supporting information.
