# Supplementary material for: Theoretical characterisation of electron tunnelling from granular activated carbon to electron accepting organisms in direct interspecies electron transfer
Source: Sci Rep. 2022 Jul 20;12:12426. doi: 10.1038/s41598-022-15606-8 (PMC9300713; doi:10.1038/s41598-022-15606-8)
Supplement: Supplementary file 1 — Supplementary Information. [file 41598_2022_15606_MOESM1_ESM.docx]

**SUPPORTING INFORMATION**

Theoretical characterisation of electron tunnelling from granular activated carbon to electron accepting organisms in direct interspecies electron transfer

Rohan Rao ^a, b^, Jing Hu ^a^, Po-Heng Lee ^a^*

1. Department of Civil and Environmental Engineering, Imperial College London, South Kensington Campus, London, UK
2. Department of Physics, Undergraduate Student, Oxford University, Oxford, UK

***Corresponding Author:**

E-mail: po-heng.lee@imperial.ac.uk

Tel: +44 (0)20 7594 5993

**A1.**

It is important to note that an electron can traverse a potential barrier by two routes: 1) classically surmounting the barrier if there is sufficient thermal energy in the system or 2) the electron quantum tunnels through the barrier. We will show later that the average potential barrier faced by the electron is approximately 0.23eV. This is sufficiently high such that the thermal energy scales of $k_{B}T\sim0.025\mathrm{eV}$ at standard temperature and pressure (STP) are too small for thermal current to be considered. Therefore, the thermal current can be neglected, and only quantum tunnelling is considered as a means of electron transfer through the potential barrier of our system.

**A2.**

Solving the diffusion equation in 3D with a gaussian function gives a root mean square distance travelled of $x_{rms}=\sqrt{6Dt}$. The self diffusion coefficient of water at ${20}^{o}C$ is $D=2.02\times{10}^{-9}m^{2}s^{-1}.$^40^ The typical length of a water molecule is 3$\dot{A}$. The average time taken for a water molecule to diffuse by 3$\dot{A}$ is therefore

$$T=\frac{\left( 3\times10^{-10} \right)^{2}}{6\times2.02\times{10}^{-9}}=7\times{10}^{-12}s$$

$$T\sim{10}^{-11}s$$

**A3.**

We postulate that there exists a continuous function $f\left( \Psi' \right)=\varphi$ that relates the submerged work function of a metal, $\Psi'$, to the potential barrier , $\varphi$, faced by an electron tunnelling out of a submerged metal through an aqueous solution. Using a Taylor expansion around $a$ gives

$$f\left( \Psi' \right)=f\left( a \right)+f^{'}\left( a \right)\left( \Psi'-a \right)+\frac{f^{''}\left( a \right)}{2}\left( \Psi'-a \right)^{2}+\ldots$$

We have the boundary condition that $f\left( \Psi_{gold}' \right)=\varphi_{gold}$ where $\Psi_{gold}'=2.26eV$ and $\varphi_{gold}=0.26eV$. Therefore setting $a=\Psi_{gold}'$ gives

$$f\left( \Psi' \right)=f\left( \Psi_{gold}' \right)+f^{'}\left( \Psi_{gold}' \right)\left( \Psi'-\Psi_{gold}' \right)+\frac{f^{''}\left( \Psi_{gold}' \right)}{2}\left( \Psi'-\Psi_{gold}' \right)^{2}+\ldots$$

Taking the limit $\Psi^{'}-\Psi_{gold}^{'}\ll1$eV gives to first order:

$$f\left( \Psi' \right)\approx f\left( \Psi_{gold}' \right)+f^{'}\left( \Psi_{gold}' \right)\left( \Psi'-\Psi_{gold}' \right)$$

Assuming that the contribution to the potential barrier is primarily due to the work function of the metal gives the boundary condition $f\left( 0 \right)=0$ which leads to

$$f^{'}\left( \Psi_{gold}' \right)\approx\frac{f\left( \Psi_{gold}' \right)}{\Psi_{gold}'}$$

Substituting

$$f\left( \Psi' \right)\approx f\left( \Psi_{gold}' \right)\frac{\Psi^{'}}{\Psi_{gold}'}$$

$$\Psi_{graphite}^{'}-\Psi_{gold}^{'}=0.16eV\ll1eV$$

So

$$f\left( \Psi_{graphite}^{'} \right)\approx f\left( \Psi_{gold}' \right)\frac{\Psi_{graphite}^{'}}{\Psi_{gold}'}$$

as stated in equation (6)
